# Supplementary material for: Beyond two alleles: multiallelic genotypic selection and its estimation from time-series data
Source: BMC Genomics. 2026 Oct 8;27:837. doi: 10.1186/s12864-026-13411-5 (PMC13651084; doi:10.1186/s12864-026-13411-5)
Supplement: Supplementary file 1 [file 12864_2026_13411_MOESM1_ESM.pdf]

Supplementary Material

Beyond two alleles: Multiallelic genotypic selection and its estimation from time-series data

Nikolas Vellnow[1], Toni I. Gossmann[1,*] and David Waxman[2,*]

[1]Computational Systems Biology,
Faculty of Biochemical and Chemical Engineering,
TU Dortmund University, Emil-Figge-Str. 66, 44227 Dortmund, Germany.

✉ nikolas.vellnow@tu-dortmund.de
https://orcid.org/0000-0002-7054-3268

✉ toni.gossmann@tu-dortmund.de, *corresponding author
https://orcid.org/0000-0001-6609-4116

[2]Centre for Computational Systems Biology,
Institute of Science and Technology for Brain-Inspired Intelligence,
Fudan University, 220 Handan Road, Shanghai 200433, People's Republic of China.

✉ davidwaxman@fudan.edu.cn, *corresponding author
https://orcid.org/0000-0001-9093-2108

In Sections 1 and 2 of this Supplementary Material we give details of estimating fitness-effect matrices from empirical frequency trajectories, including an additional example illustrating a case where the assumption of constant selection is poorly supported. In Section 3 we explore how trajectory length, genetic drift, and the ridge-penalty hyperparameters influence the estimate of the fitness-effect matrix. In Section 4 we provide simulation-based validation of our empirical yeast findings, including a comparison against a neutral, drift-only null model and an assessment of the reliability of our heterozygote-advantage classification. In Section 5 we explore the sensitivity of the number of alleles detected at a locus to the level of filtering applied to pooled sequencing data, and in Section 6 we provide calculations and simulations to illustrate detection and threshold effects.

1 Details of estimating the $\mathbf{F}$ matrix from empirical data

For the estimation of $\mathbf{F}$ with our 'cost function' $C(\mathbf{F})$ (see Eq. 53 in Appendix D.2), we used the 'fmincon' optimisation function in MATLAB R2026a (The Mathworks Inc., 2025), which

implements gradient-based methods for constrained minimization. This method performed well, especially in combination with a ridge regularization with a $\lambda = 6 \times 10^{-3}$, which penalizes very extreme and thereby unlikely fitness values.

2 Estimating F matrix from empirical data: Another yeast example

As another example, we also estimated $\mathbf{F}$ (with constrained minimization) for trajectories of the haplotypes in the 10 KB window around position 436,424 on chromosome C12, for replicate population 3 (see Figure S1a), from the data set of Burke, Liti, and Long (2014) and Phillips et al. (2020). The estimated $\mathbf{F}$ and the corresponding matrix of relative fitnesses $\mathbf{w}$ (minimum cost: 1.276) are:

$$\mathbf{F} = \begin{pmatrix} 0.000\,, -0.053\,, & 0.861\,, -0.007 \\ -0.053\,, -0.415\,, & 0.713\,, \quad 0.232 \\ 0.861\,, \quad 0.713\,, & -0.188\,, \quad 0.076 \\ -0.007\,, \quad 0.232\,, & 0.076\,, -0.305 \end{pmatrix}, \qquad \mathbf{w} = \begin{pmatrix} 0.537\,, 0.509\,, 1.000\,, 0.534 \\ 0.509\,, 0.314\,, 0.921\,, 0.662 \\ 1.000\,, 0.921\,, 0.436\,, 0.578 \\ 0.534\,, 0.662\,, 0.578\,, 0.374 \end{pmatrix}. \tag{S1}$$

The estimated $\mathbf{F}$ leads to trajectories that resemble the empirical ones, although the resemblance is not very strong (see Figure S1b). In particular, we hypothesize that after generation 9 the selective forces may have changed; since under that scenario $\mathbf{F}$ would not have been constant, our estimation method may not find a well-matching $\mathbf{F}$, consistent with this window's comparatively high cost value of 1.276, which lies above the 75th percentile (0.785) of the cost-value distribution across all $1,161$ yeast windows. More broadly, this distribution is tightly concentrated at low values for the majority of windows (median 0.550; interquartile range $[0.421,\, 0.785]$), but has a long right tail (mean 1.217; 95th/99th percentiles $4.716/12.870$). This indicates that while most windows are well described by a constant $\mathbf{F}$, a minority – such as the window around position 436,424 on chromosome C12 – show an elevated cost, plausibly reflecting genuine violations of the constant-selection assumption rather than a general shortcoming of the estimation procedure.

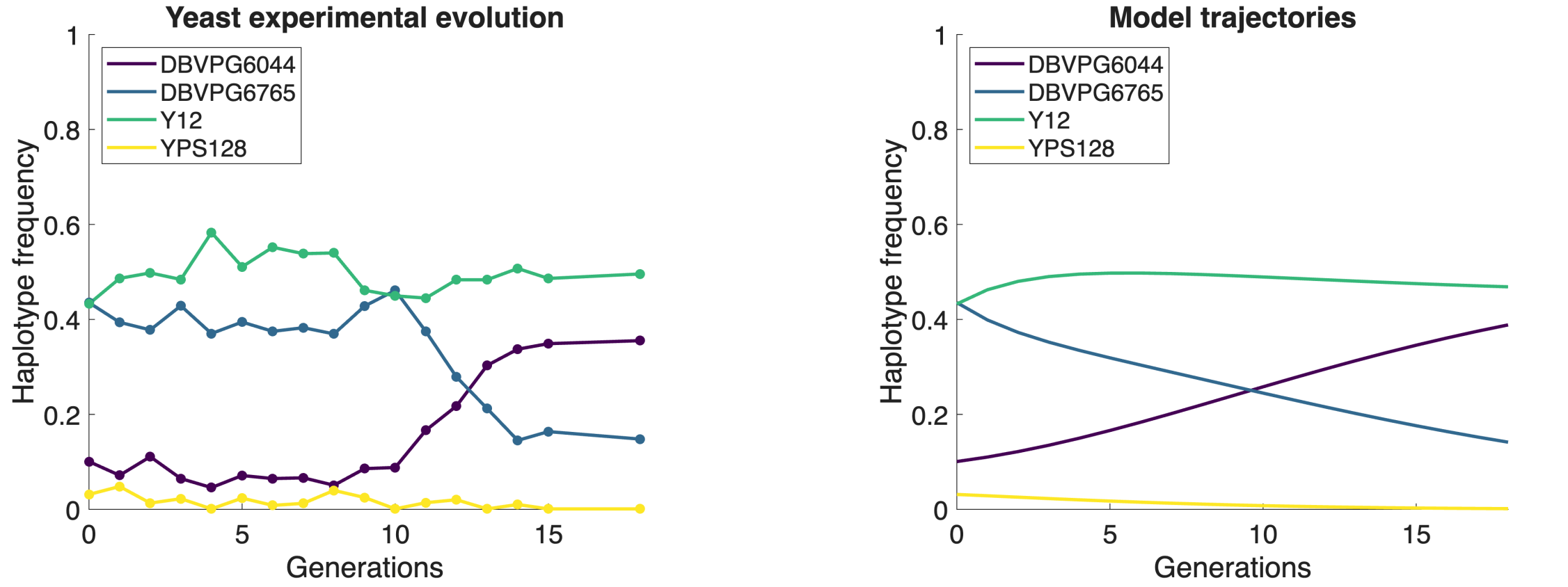

(a) Empirical haplotype frequencies from yeast data.

(b) Trajectories modelled based on the estimated $\mathbf{F}$ matrix (right).

Figure S1: Estimating the $\mathbf{F}$ matrix from brewer's yeast haplotype frequency trajectories. Data is from a 10 KB window around position 436,424 on chromosome C12 for replicate 3 (Burke, Liti, and Long, 2014; Phillips et al., 2020). Haplotypes are DBVPG6044, West African in dark blue, DBVPG6765, wine/European in purple, Y12, sake/Asian in green, and YPS128, North American in yellow.

3 Performance of the inference method across population sizes and trajectory lengths

In order to investigate how the length of allele frequency trajectories affects the estimate of $\mathbf{F}$ and $\mathbf{w}$ correspondingly, we proceeded as follows.

We generated trajectories based on a known $\mathbf{F^{(0)}}$. Using trajectories produced in this way, but of different length (i.e., different time duration), yielded different estimates of the $\mathbf{F}$ matrix. We compared the estimates with the actual fitness effect matrix, namely $\mathbf{F^{(0)}}$.

We began by generating an $\mathbf{F^{(0)}}$ with elements, drawn from a normal distribution with mean 0 and standard deviation 0.1, along with random initial allele frequencies. From these, we simulated trajectories with varying population sizes ($N = \infty$, $N = 10^6$, $N = 10^5$ and $N = 10^4$) of length 40 generations. We next used our method to estimate $\mathbf{F}$ and $\mathbf{w}$, based on trajectories of different lengths (corresponding to different experimental observation periods), ranging from 1 to 40 generations. Next, we calculated, for each estimated $\mathbf{w}$ matrix, the distance to the true relative fitness matrix (namely $\mathbf{w^{(0)}}$), along with the corresponding Spearman rank correlation coefficient.

In Appendix D.1 we introduced the $\mathbf{v}$ vector, which contains the independent elements of the matrix of fitness effects. The distance was calculated as the mean absolute error (MAE)

between entries of the true $\mathbf{w}^{(\mathbf{0})}$ and the estimated $\mathbf{w}$:

$$\mathsf{MAE}(\mathbf{v}_{\mathsf{opt},w}, \mathbf{v}_{0,w}) = \frac{1}{n} \left\| \mathbf{v}_{\mathsf{opt},w} - \mathbf{v}_{0,w} \right\|_1 \tag{S2}$$

where $\|\cdot\|_1$ represents the ℓ_1- norm, and $\mathbf{v}_{0,w}$ and $\mathbf{v}_{\mathsf{opt,w}}$ represent the true and the estimated vector $\mathbf{v}$, respectively, when transformed to relative fitness values, and n represents their length.

Finally we repeated this procedure for 256 independently generated $\mathbf{F}^{(\mathbf{0})}$ matrices. From these matrices, for each of the different observation lengths, we calculated the median, along with the 25% and 75% quantiles of MAE.

We observe good convergence of the distance and correlation measures within 40 generations (see Figure S2, row 1-2). A substantial part of the improvement in the $\mathbf{w}$-estimation occurs within the first 10 generations and a very well estimated $\mathbf{w}$ is reached after 30 generations (see Figure S2, row 1). The rank ordering of estimated fitness values correlates strongly with the true ordering after about 10 generations and exceeds the null expectation after roughly 30 generations (see Figure S2, second row). This suggests that statements about the rank ordering of estimated fitness values of different genotypes will typically be correct after relatively short observation periods. By contrast, the error of the precise values of $\mathbf{w}$ are more influenced by stochasticity introduced by genetic drift, but are expected to be smaller than 0.03 even for rather small populations like $N = 10^4$.

Furthermore, we also simulated trajectories for the same set of population sizes, but under neutral evolution, where all genotypes had the same fitness and used our method to estimate $\mathbf{w}$. We then calculated the mean relative fitness for each estimated $\mathbf{w}$ in replicate trajectories and plotted the change over time and the distribution at the end of 40 generations (Figure S2, row 3 and 4, respectively). If perfectly estimated, the mean relative fitness should be 1.0. As can be seen from Figure S2, row 3 the estimated values lie close to the true value. Comparing the mean relative fitness inferred from real data with the 5th percentile of this null distribution provides a simple statistical test for rejecting the hypothesis of neutral evolution.

Furthermore, we also simulated trajectories for the same set of population sizes, but under neutral evolution, where all genotypes had the same fitness, and used our method to estimate $\mathbf{w}$. For each replicate trajectory, we then calculated the mean of the entries of the estimated $\mathbf{w}$, denoted $\bar{w}$, and plotted its change over time and its distribution at the end of 40 generations (Figure S2, row 3 and 4, respectively). If perfectly estimated, $\bar{w}$ should be 1.0. As can be seen from Figure S2, row 3, the estimated values lie close to the true value. Comparing $\bar{w}$ inferred from real data with the 5th percentile of this null distribution provides a simple statistical test for rejecting the hypothesis of neutral evolution.

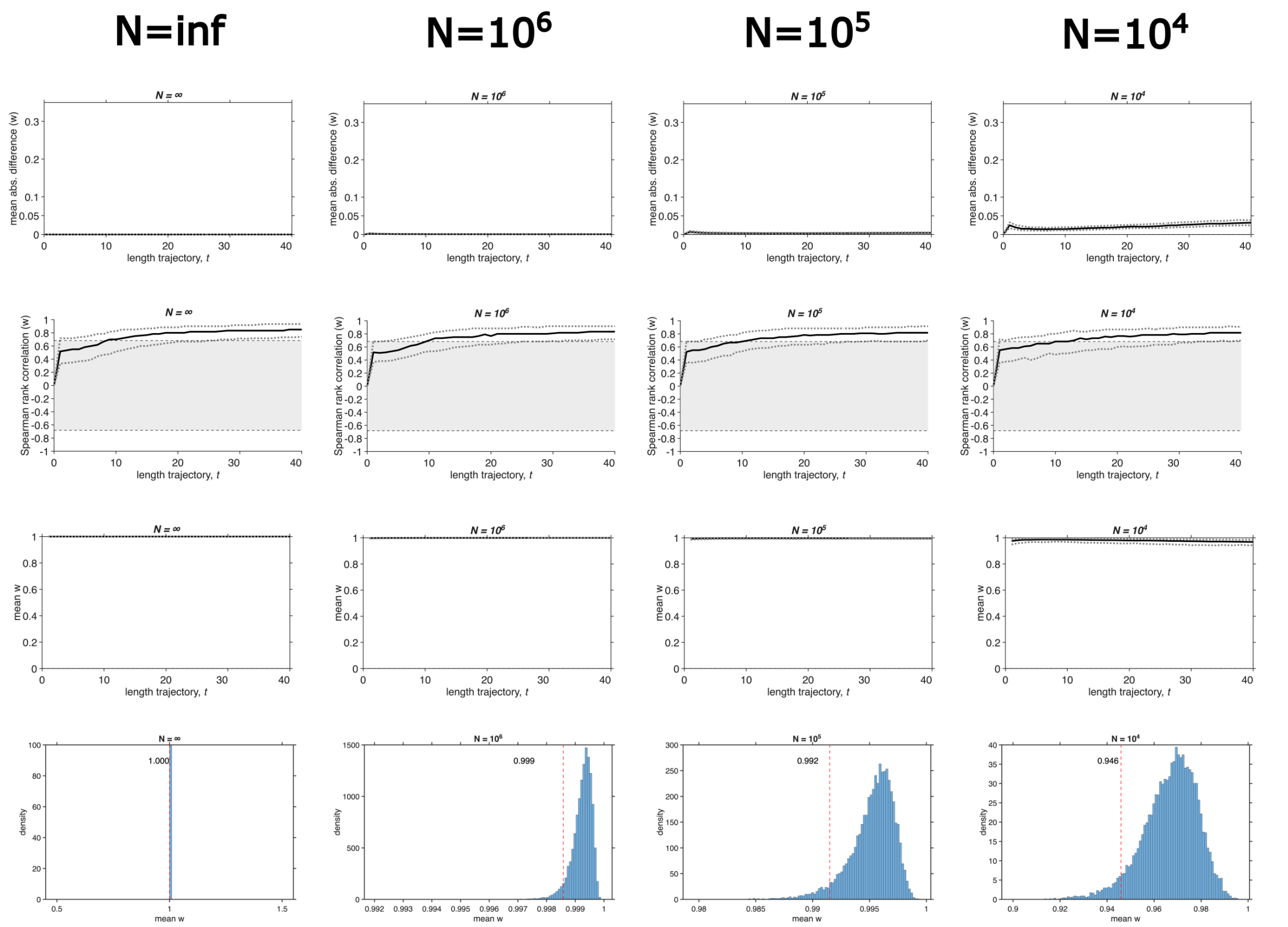

Figure S2: Performance of the inference method across population sizes and trajectory lengths. Columns correspond to population size N, and rows show different summary measures or selection scenarios. For the first three rows, the x-axis indicates the length of the observed trajectory. The first row shows the mean absolute difference between the true and the estimated relative fitness matrix. The second row shows the Spearman rank correlation coefficient between the true and estimated $\mathbf{w}$ matrices; the grey shaded region indicates the 95% confidence interval expected for the correlation between two random matrices (null expectation). The third row shows $\bar{w}$, the mean relative fitness per estimated matrix under neutral evolution, representing the method's null expectation in the absence of selection. In rows 1–3, the black line denotes the median across 256 replicate simulations and the dotted lines indicate the corresponding percentile ranges (25–75% in rows 1–2, 2.5–97.5% in row 3). The bottom row shows the distribution of $\bar{w}$ after 40 generations of neutral evolution across 16,384 replicate simulations; the dashed red line marks the 5th percentile, below which $\bar{w}$ values are unlikely under neutrality.

Sensitivity of estimation to the ridge-penalty hyperparameters α and λ

The cost function used for estimating $\mathbf{F}$ (Eq. 48 in Appendix D) includes two hyperparameters: α, a small constant added to the denominator to avoid infinite weightings at vanishing allele frequencies, and λ, the ridge-penalty weight that shrinks estimated fitness effects towards zero. In the main text we used $\alpha = 10^{-6}$ and $\lambda = 6 \times 10^{-3}$, values chosen based on empirical performance. Here we assess the sensitivity of estimation accuracy to these choices systematically, and across population size.

For each combination of $\alpha \in \{10^{-8}, \ldots, 10^{-2}\}$ (7 log-spaced values) and $\lambda \in \{10^{-8}, \ldots, 10^{-1}\}$ (24 log-spaced values), and for each of three population sizes ($N = 10^3$, 10^4, 10^6), we generated $n_{\text{rep}} = 64$ independent replicate simulations. In each replicate, we constructed a random 4-allele fitness matrix $\mathbf{F}^{(0)}$ by drawing its independent entries i.i.d. from a normal distribution (mean 0, SD 0.1), and simulated a population of the given size, starting from random initial allele frequencies, evolving under this true $\mathbf{F}^{(0)}$ for $T = 100$ generations. For each replicate, we then estimated $\mathbf{F}$ (and the corresponding relative fitness matrix $\mathbf{w}$) from the full simulated trajectory, using the given (α, λ) pair, via the same multi-start constrained optimization procedure used elsewhere ($n_{\text{starts}} = 80$).

For each replicate we computed two measures of estimation accuracy, applied to $\mathbf{w}$: the Spearman rank correlation between the true and estimated relative fitness vectors, and the mean absolute error (MAE) between them (as defined in Eq. S2). We then summarized these measures, at each (α, λ) combination and population size, by their mean across the 64 replicates, yielding six 7×24 grids of estimation accuracy across the hyperparameter space – one pair of grids (rank correlation, MAE) per population size (Figure S3).

We observe little sensitivity to changes in α across the analysed values; performance instead depended primarily on λ, and the best-performing λ decreased with population size, from $\lambda \approx 5 \times 10^{-2}$ at $N = 10^3$ to $\lambda \approx 3.7 \times 10^{-4}$ at $N = 10^6$. The default values used in the main text ($\alpha = 10^{-6}$, $\lambda = 6 \times 10^{-3}$) fall within a region of comparably good performance at all three population sizes, and can, therefore, be considered a good compromise in situations where the precise population size is unknown.

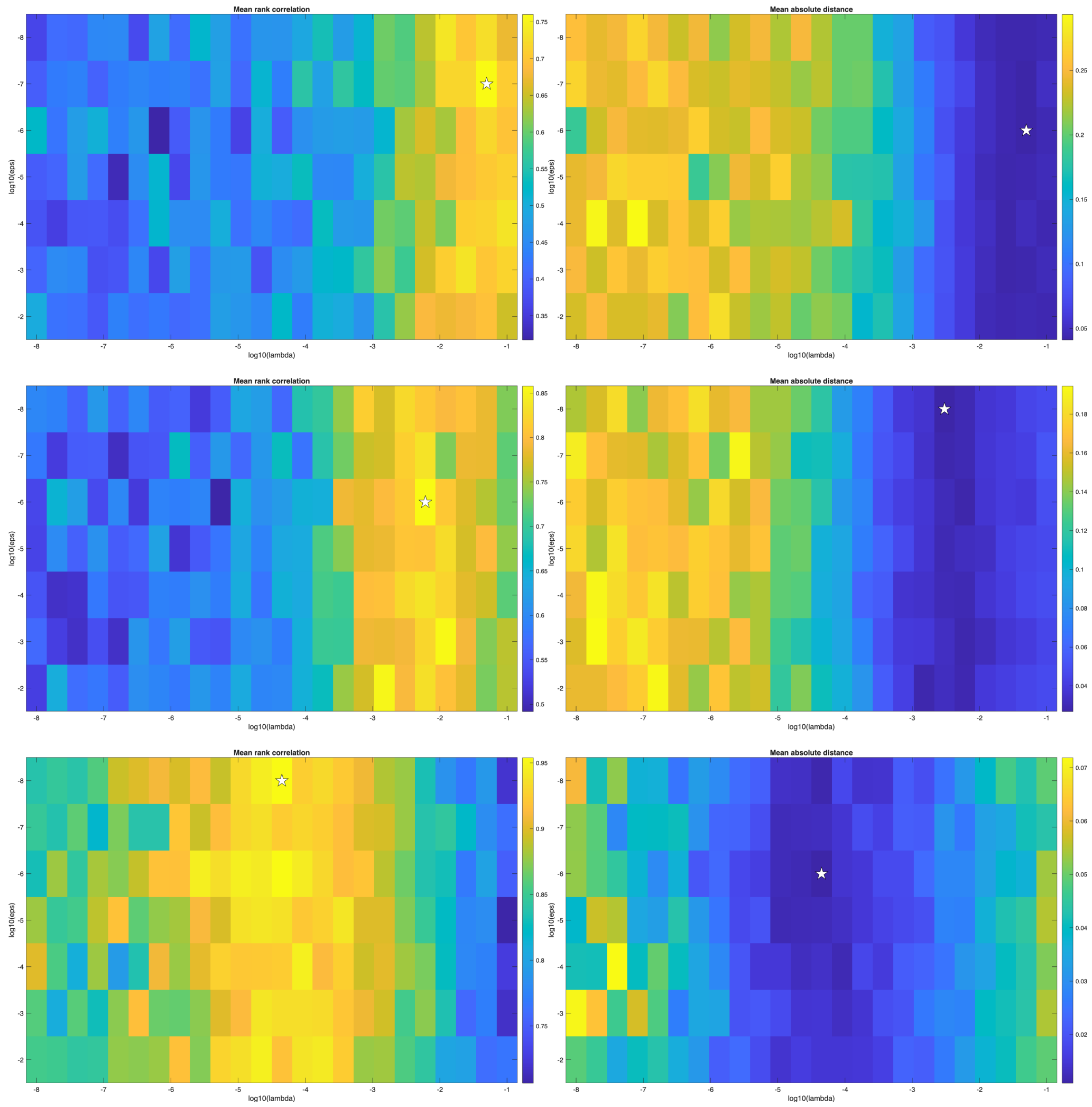

Figure S3: Estimation accuracy is robust across a wide range of the ridge-penalty hyperparameters α and λ, across population sizes. Heatmaps show estimation accuracy across a grid of α (7 log-spaced values, 10^{-8} to 10^{-2}) and λ (24 log-spaced values, 10^{-8} to 10^{-1}), summarized across $n_{\text{rep}} = 64$ replicate simulations per (α, λ) combination, at $N = 10^3$ (top row), $N = 10^4$ (middle row), and $N = 10^6$ (bottom row). Left column: mean Spearman rank correlation between the true and estimated relative fitness vectors. Right column: mean absolute error (MAE) between the true and estimated relative fitness vectors. In all heatmaps, colour indicates the value of the corresponding metric, from dark blue (low) to yellow/orange (high); since high rank correlation and low MAE both indicate good estimation accuracy, yellow/orange denotes good performance in the left column, while dark blue denotes good performance in the right column. In each panel, the white star marks the (α, λ) combination that performed best by the corresponding metric.

4 Validation of yeast findings with simulated data

Non-neutral evolution

To test whether neutral evolution due to genetic drift could cause relative fitness values as high or higher than we have found for the yeast data, we simulated evolutionary trajectories for relatively low population sizes ($N = 10^3$, 10^4) and compared the results with the empirical yeast data. We set the true 4×4 fitness matrix $\mathbf{F}^{(0)}$ to be exactly neutral (all entries zero), so that no true fitness differences existed among the genotypes. For each of $n_{\textsf{rep}} = 2^{14} = 16{,}384$ independent replicate simulations at each population size, we drew random initial allele frequencies and simulated genotype frequencies forward for $T_{\max} = 40$ generations under Hardy–Weinberg random mating and viability selection governed by $\mathbf{F}^{(0)}$. Since $\mathbf{F}^{(0)} = \mathbf{0}$, selection left genotype frequencies unchanged at every generation, so any change in allele frequencies arose purely from genetic drift – implemented via multinomial sampling of N diploid individuals' genotypes each generation – rather than from true fitness differences. We then applied the same estimation procedure used for the empirical yeast data and the random-$\mathbf{F}^{(0)}$ simulations described below (multi-start constrained optimization of the ridge-penalized cost function; $n_{\textsf{starts}} = 80$, $\lambda = 0.0061$) to each trajectory, yielding an estimated fitness matrix $\mathbf{F}$, which we converted to the relative fitness matrix $\mathbf{w}$ (as defined above) and summarized via its mean $\bar{w}$: values close to 1 indicate near-uniform estimated fitness, as expected under true neutrality, while low values indicate spuriously inferred fitness differences. Repeating this across all replicates yielded, for each population size, a null distribution of $\bar{w}$ attributable entirely to genetic drift and estimation noise, against which we compared the corresponding empirical distribution of $\bar{w}$ across the yeast genomic sites (computed identically from each site's estimated $\mathbf{F}$).

At $N = 10^4$, the null distribution had a median $\bar{w}$ of 0.968 (5% quantile: 0.946); at $N = 10^3$, drift alone pushed the null median down to 0.865 (5% quantile: 0.796), reflecting the expected increase in drift at smaller population size. The empirical median $\bar{w}$ across yeast sites was 0.644 – substantially lower than either null distribution, including the more permissive $N = 10^3$ case (Figure S4). Correspondingly, the large majority of empirical sites fell below the lower 5% quantile of the null distribution at both population sizes: 99.57% (1156 of 1161 sites) at $N = 10^4$, and 98.54% (1144 of 1161 sites) at $N = 10^3$.

This indicates that the pronounced fitness heterogeneity observed among yeast genotypes is highly unlikely to be explained by genetic drift alone. Note that our cost function already incorporates a large-N approximation to the sampling likelihood under drift, via the Shahshahani metric, and the population sizes used here ($N = 10^3, 10^4$) are well below the yeast experiment's reported effective population size ($N_e \approx 10^6$; see the more detailed discussion of this quantity

in the following subsection), providing a conservative test of this approximation under stronger drift than is likely present in the real data.

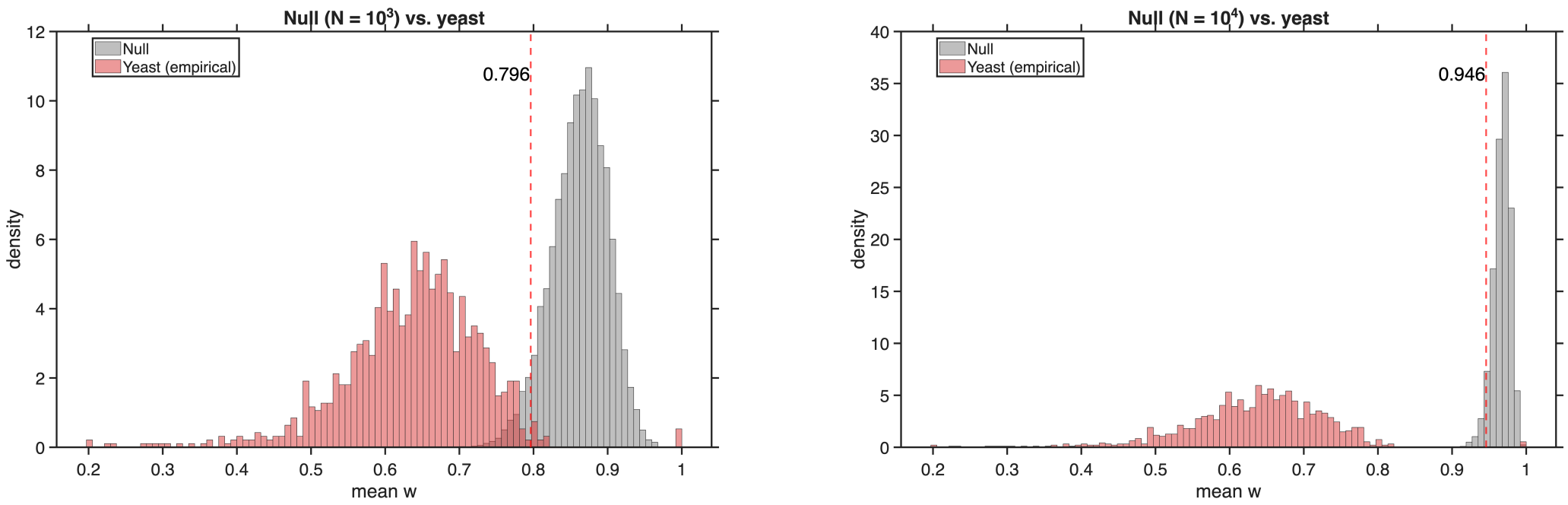

Figure S4: Empirical yeast fitness heterogeneity exceeds the neutral null expectation at both population sizes. Distributions of the mean relative fitness $\bar{w}$ under simulated neutral evolution (grey; $n = 16{,}384$ replicates per panel) compared to the empirical distribution of $\bar{w}$ across the 1161 yeast genomic sites (red), at $N = 10^3$ (left) and $N = 10^4$ (right). The dashed red line marks the 5th percentile of the null distribution in each panel. The empirical yeast distribution lies almost entirely below this threshold at both population sizes (98.54% and 99.57% of sites at $N = 10^3$ and $N = 10^4$, respectively), indicating that the fitness heterogeneity observed in yeast is highly unlikely to arise from genetic drift alone.

Heterozygote advantage

To test whether our estimation method could itself produce a signal of heterozygote advantage that is not actually present in the underlying fitness matrix—and how reliably it detects heterozygote advantage when it is present—we simulated evolutionary trajectories in MATLAB and applied our estimation procedure to the simulated data.

For each of three population sizes ($N = 10^3$, 10^4, 10^5), we independently generated $2^{14} = 16{,}384$ replicate simulations (a number chosen for computational convenience). In each replicate, we constructed a random 4-allele fitness matrix $\mathbf{F}^{(\mathbf{0})}$ by drawing its entries from a normal distribution (mean 0, SD 0.1); one diagonal entry is fixed at 0 as a reference allele. We then simulated a population with four alleles, starting from random initial frequencies, evolving under this true $\mathbf{F}^{(\mathbf{0})}$ for 40 generations, and applied our estimation procedure to the full simulated frequency trajectory to obtain an estimated $\mathbf{F}$.

To assess the reliability of this result, we repeatedly subsampled from the pool of $16{,}384$ replicates. For each population size, we drew 1000 independent samples, each consisting of 1161 replicates chosen at random without replacement (i.e., no replicate is counted twice within a given sample). Because each of the 1000 samples is drawn afresh from the full pool of $16{,}384$, the same replicate can appear in multiple samples. Sample size (1161) was chosen to match

the number of analysed genomic windows in the yeast experiment. Within each sample, we split the replicates into two groups based on the true, simulated $\mathbf{F}^{(\mathbf{0})}$: replicates where the most-fit genotype was a homozygote, and replicates where it was a heterozygote. We then computed, within each group, the proportion of replicates where the estimated $\mathbf{F}$ also indicated a heterozygote as most-fit. This yields, for each population size, both a false-positive rate (spurious heterozygote calls when the truth is homozygous) and a true-positive rate (correct heterozygote calls when the truth is heterozygous) for our estimation procedure.

At every population size tested, our procedure showed substantial discriminative power between true heterozygote advantage and its absence: a heterozygote call was 1.9–3.9 times more likely when the true most-fit genotype was genuinely a heterozygote than when it was a homozygote (positive likelihood ratio TPR/FPR: 1.86, 3.28, and 3.87 at $N = 10^3$, 10^4, and 10^5, respectively). This discriminative power increased with population size, driven primarily by a false-positive rate that fell sharply with N – from 0.508 (95% CI $[0.459, 0.556]$) at $N = 10^3$ to 0.278 (95% CI $[0.233, 0.323]$) at $N = 10^4$ and 0.232 (95% CI $[0.191, 0.276]$) at $N = 10^5$ – while the true-positive rate remained consistently high throughout, at 0.945 (95% CI $[0.930, 0.960]$), 0.912 (95% CI $[0.892, 0.932]$), and 0.897 (95% CI $[0.876, 0.918]$), respectively (Figure S5). The effective population size of $N_e \approx 10^6$ reported for this yeast experiment reflects a bottleneck size deliberately imposed at two separate points within each 30-generation cycle between forced outcrossing events – once several generations after mating, and again mid-way through the subsequent liquid-culture growth phase – with substantial population expansion (to stationary-phase densities of 0.8–4×10^9 cells/ml) occurring between bottlenecks (cf. Fig. S1 of Burke, Liti, and Long, 2014). Consequently, $N_e \approx 10^6$ does not correspond directly to the population size at a single, uniform generation in our simulations, which model one full cycle as a single discrete generation. Instead, the effective population size relevant to genetic drift over such a cycle depends on the bottleneck size, the number of bottlenecks per cycle, and the extent of intervening growth, and cannot be recovered from the bottleneck size alone. We therefore do not attempt to map $N_e \approx 10^6$ onto a specific point in our simulated range, noting only that our tested population sizes ($N = 10^3$–10^5) are comparable to or smaller than the bottleneck size itself, giving some indication of expected performance under repeated strong bottlenecks. This makes it unlikely that the high prevalence of heterozygote advantage observed in the yeast data is primarily driven by estimation artifacts.

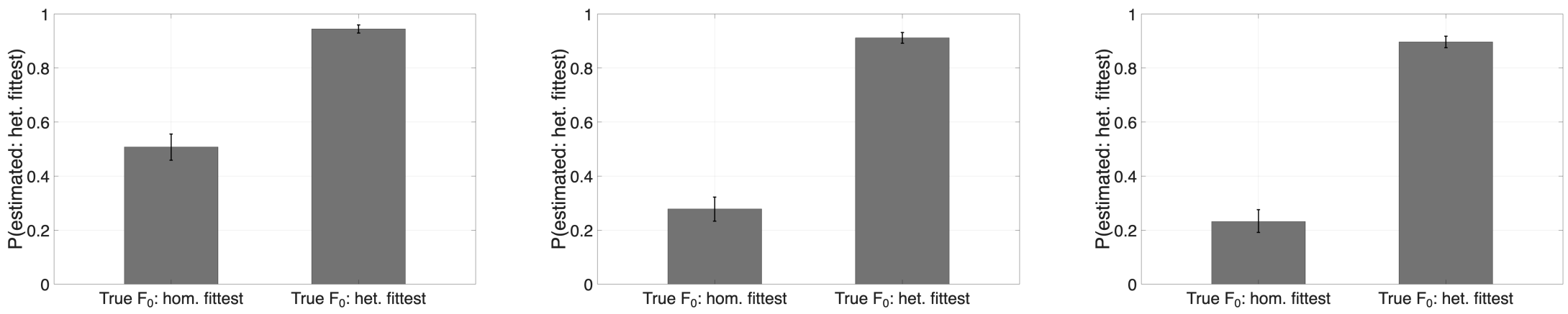

Figure S5: The estimation procedure discriminates heterozygote advantage from its absence, though with a non-negligible false-positive rate at small population size. For each population size ($N = 10^3$, 10^4, 10^5; left to right), replicates were split by the true identity of the most-fit genotype, and we computed the proportion of replicates for which the estimated $\mathbf{F}$ also called it a heterozygote. The left bar is thus a false-positive rate; the right bar a true-positive rate. The false-positive rate decreases with population size ($0.508 \to 0.278 \to 0.232$) while the true-positive rate remains consistently high ($0.945 \to 0.912 \to 0.897$). Error bars denote 95% CI over subsamples (see main text).

Both analyses in this section rely on subsampling procedures that treat the $1{,}161$ replicates within each simulated sample as statistically independent. The corresponding empirical genomic windows, however, are not independent: as discussed in the main text, adjacent 10 KB windows may retain substantial linkage, since sexual reproduction in the yeast experiment occurred only once every 30 generations. The effective number of independent observations underlying the empirical comparisons is therefore likely smaller than $1{,}161$, and the null distributions and confidence intervals reported above should be interpreted as characterising sampling variability under an idealised independence assumption, rather than as fully accounting for the dependence structure among linked genomic windows.

5 Extracting multiallelic trajectories from pooled sequencing data

Multiallelic variation can be identified from next-generation sequencing (NGS) data using appropriate variant calling and filtering strategies. This includes the detection of small haplotypes (combinations of nearby SNPs) and structural variation, as well as estimation of allele frequencies in pooled sequencing samples. To illustrate this, we analyzed pooled sequencing data from a population of *Drosophila melanogaster* that was sampled at multiple time points. Variant calling was performed using FreeBayes (Garrison and Marth, 2012), which supports the identification of complex haplotypes and structural variants. Our analysis focused on chromosome arm 3R across three distinct sampled time points (July 2009, July 2012, and October 2015) from the "Linvilla" population, which is part of the "Drosophila in Time and Space" (DEST) project (Kapun et al., 2021). We applied a stringent filter, retaining only sites where each detected allele was supported by at least three reads at every sampled time point. This approach uncovered a

wide range of multiallelic variation: we found 17,678 variant sites with three alleles, 1,602 with four, 180 with five, 14 with six, and 2 with seven alleles (see Figure S6). Considerable numbers of multiallelic variants were also found when we used different filtering thresholds (see Figure S7). We provide the code for this extraction pipeline in our github repository `https://github.com/NikolasVellnow/selection_multiallelic`.

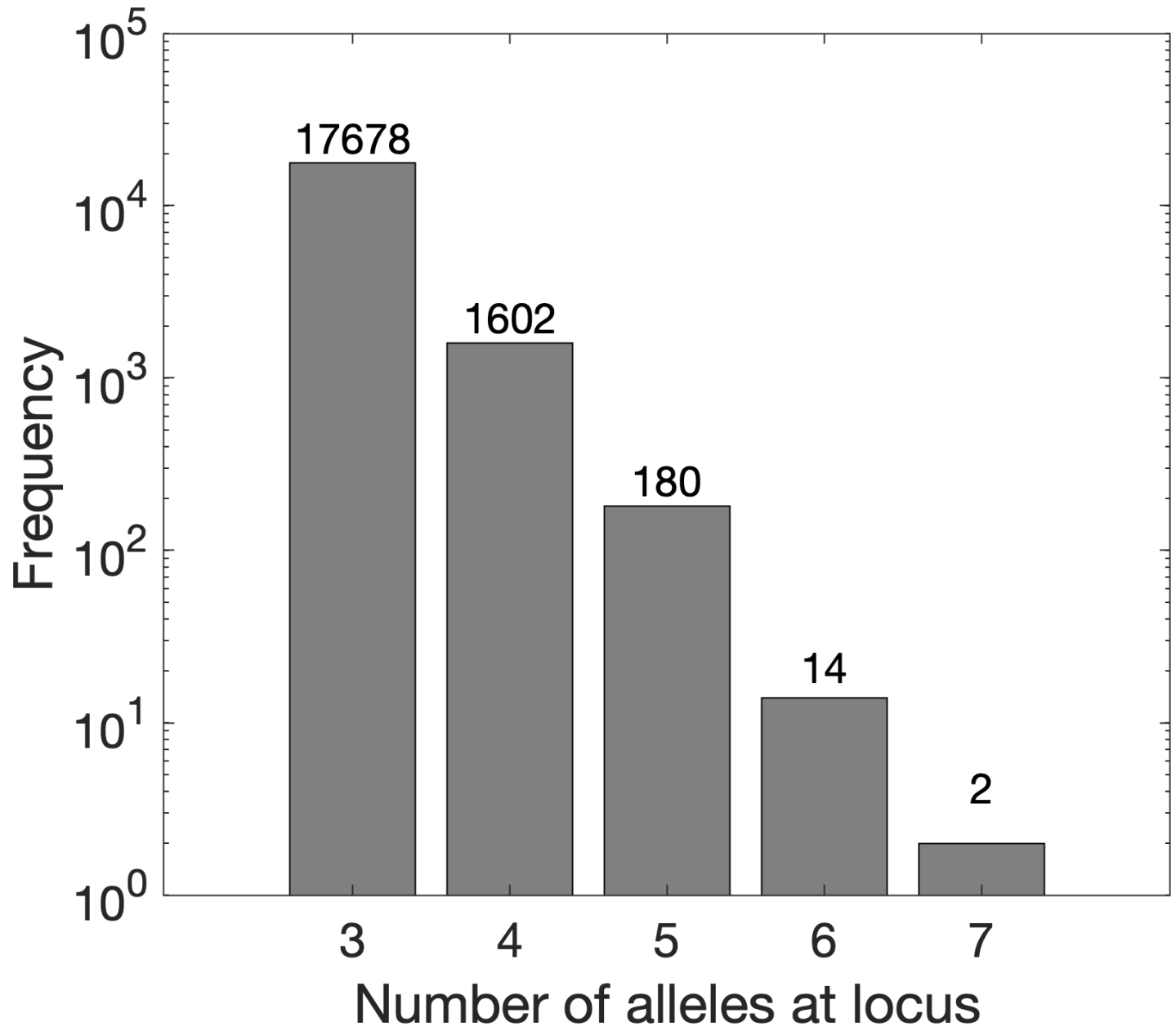

Figure S6: The number of sites that harbor more than two alleles in a natural D. melanogaster population. Data are shown for the chromosome arm 3R of the 'Linvilla' population obtained for three different time points (July 2009, July 2012, and October 2015), which is part of the DEST dataset (Kapun et al., 2021). We included only sites for which each allele was supported by at least three reads at each of the three sampled time points, i.e. we applied a hard filter.

Whether an allele is considered present depends on the filtering criteria applied to sequencing reads. In pooled sequencing data, a common approach is to require that each allele be supported by a minimum number of reads. In our primary analysis we required at least three reads supporting each allele at every sampled time point. Varying this threshold changes the number of detected multiallelic sites (see Figure S7). However, even under more stringent thresholds (e.g. requiring ≥ 7 reads per allele), substantial numbers of sites with three or four alleles remain detectable.

These results demonstrate that multiallelic polymorphisms are common in pooled sequencing data and can provide suitable trajectories for the inference framework developed in this study.

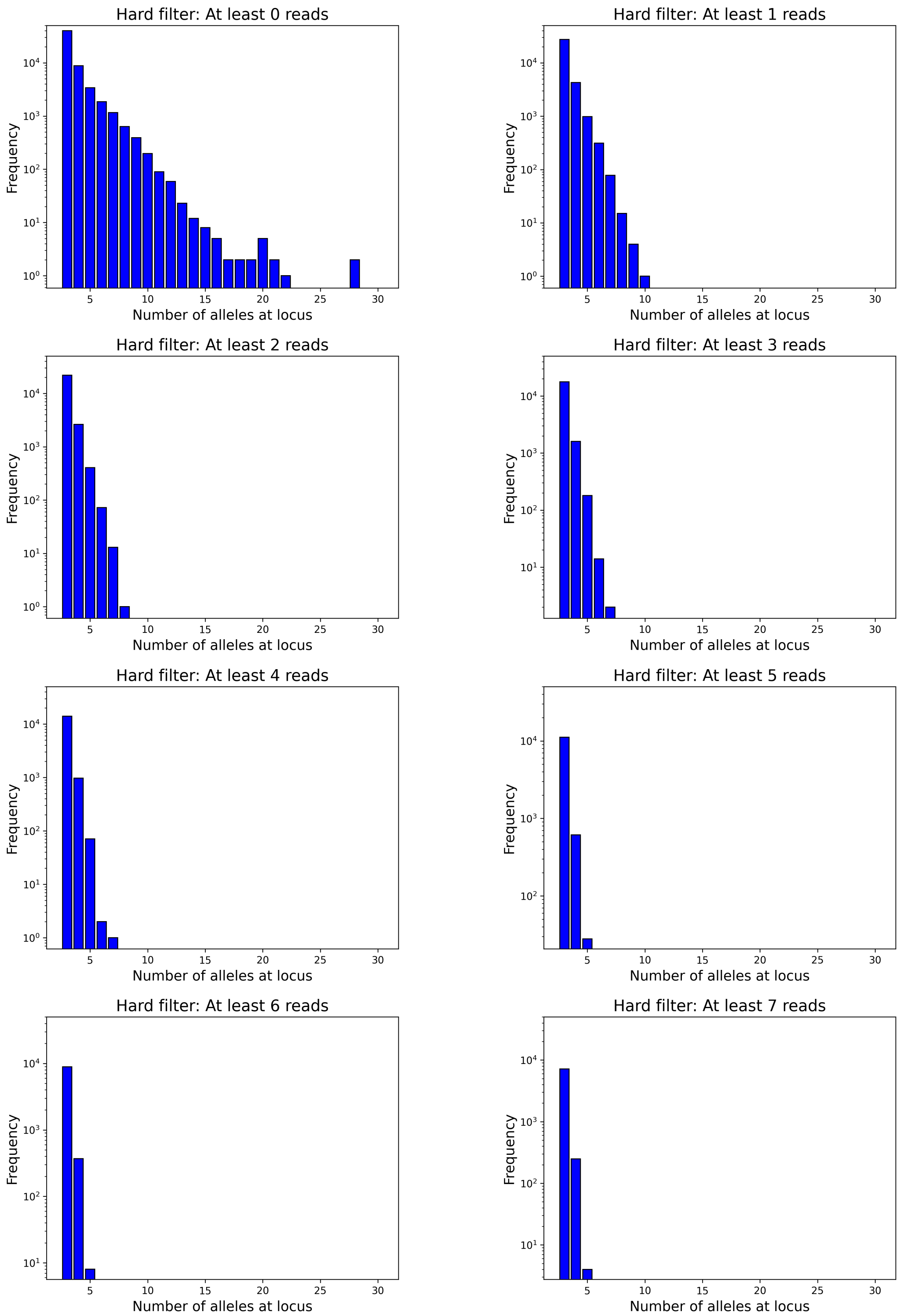

Figure S7: Sensitivity of number of alleles on hard filtering. Histograms are shown for the number of sites harbouring different numbers of alleles. The only sites included were those in which all alleles/haplotypes were supported by at least the specified minimum number of reads, at each of the three time points. This filtering criterion, for the minimum number of reads, was varied in the different subplots.

6 Probability of detection and thresholds

Here we provide calculations and simulations to substantiate some of the arguments given in the Discussion of the main text, concerning detection and threshold effects.

We used MATLAB for this purpose.

First, we modelled the probability of detection, for a coverage of x, as 1 minus the probability that the allele will not be contained in any of the x sequenced reads, i.e., probability of detection $= 1 - (1 - p)^x$, where p is the relative frequency of the allele. We then plotted the probability of detection against the coverage for several values of p (see Figure S8a). Second, we simulated evolution of a locus with ten alleles, with a randomly generated $\mathbf{F}$ matrix. We then imitated the effect that a detection threshold would have on the allele frequency trajectories to be analyzed (see Figure S8b).

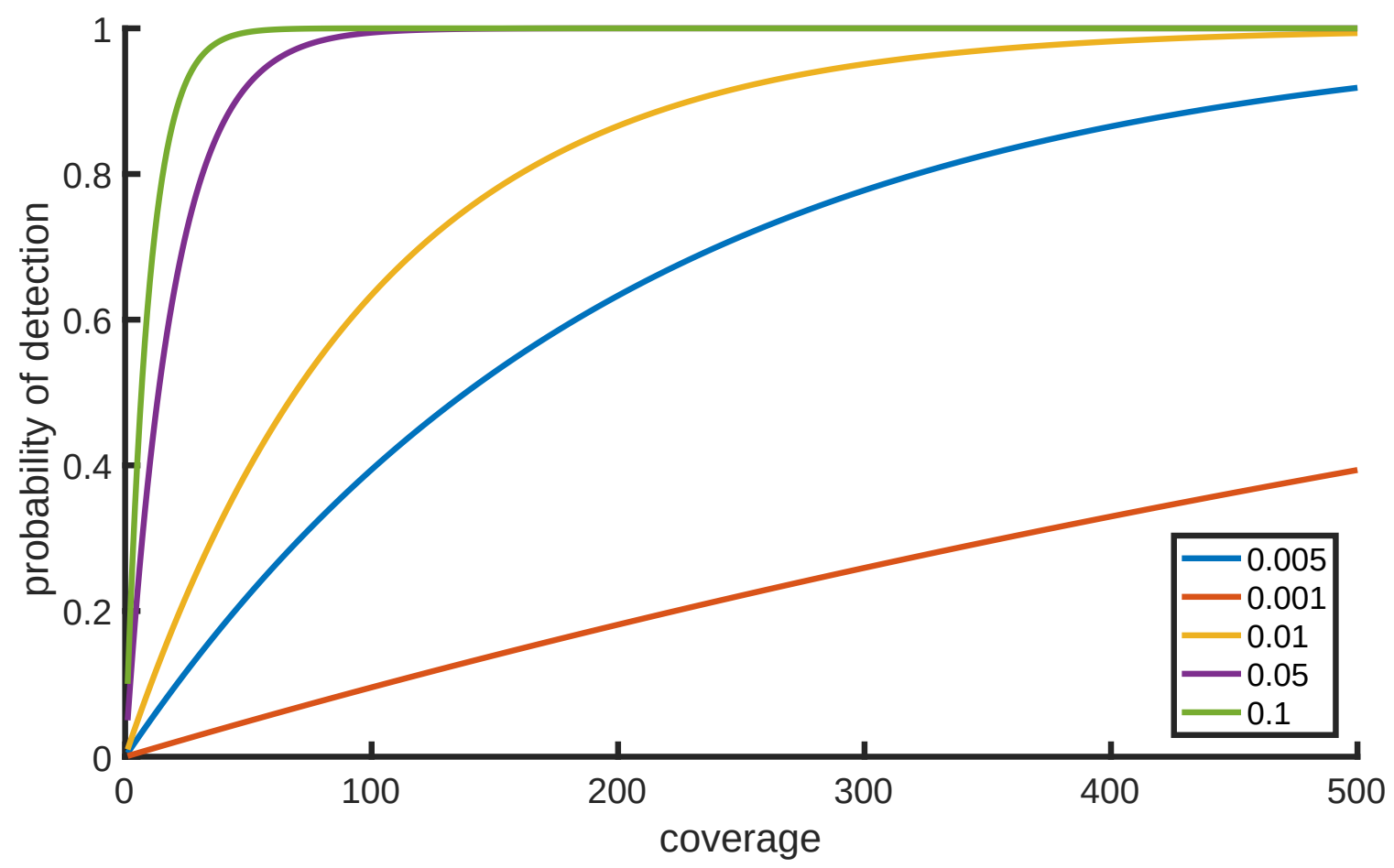

(a)

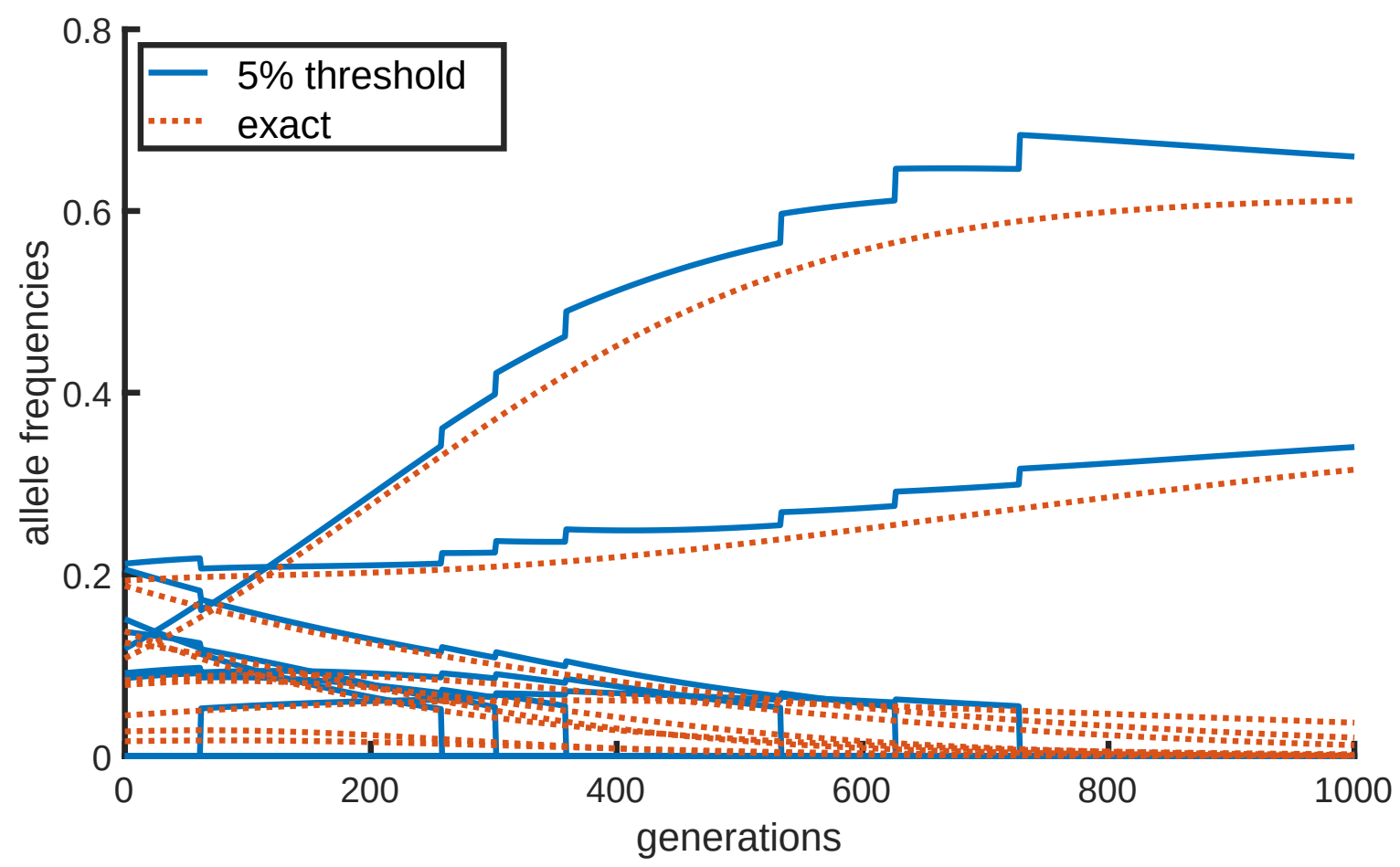

(b)

Figure S8: Illustration of observation thresholds in the analysis of multiallelic trajectories. (a) Probability to detect a genetic variant at a given frequency assuming different levels of sequencing coverage, as applied to pooled samples. (b) Plots of the exact allele frequency trajectories (red), and trajectories after applying a 5% threshold (blue) for a locus with ten alleles and an arbitrarily chosen scheme of selection. The threshold: (i) sets allele frequencies below 5% to zero, and (ii) normalizes the remaining frequencies so that they sum to unity. A consequence of (i) and (ii) are the 'steps' in the blue curves.
